# News about medicine affect compliance in people with hypochondriac beliefs

**DOI:** 10.1192/j.eurpsy.2021.1252

**Published:** 2021-08-13

**Authors:** S. Kumchenko, O. Shekina, E. Rasskazova, A. Tkhostov

**Affiliations:** 1 Clinical Psychology, Moscow State University, Moscow, Russian Federation; 2 Faculty Of Psychology, Lomonosov MSU, Moscow, Russian Federation

**Keywords:** hypochondriac beliefs, compliance

## Abstract

**Introduction:**

The context of infodemic and necessity of preventive behavior (Roy et al., 2020) demands for studies of the role of news in compliance including health priming (Gibbons, 2003, Pechmann, 1999). Especially important is a topic the effect of news about traditional and alternative medicine (Furnham, Forey, 1994) their impact on subjective compliance.

**Objectives:**

The aim was to study the relationship to medicine and subjective compliance in people with hypochondriac beliefs after priming by negative news about traditional and alternative medicine.

**Methods:**

122 healthy adults (56 males, mean age 40.7±13.6) were randomized to conditions (control, negative news about traditional medicine, negative news about alternative medicine); then they read and appraised four news (in two experimental groups one of them was about medicine); filled changes in emotions after reading (PANAS, Carver et al., 1989), Cognitions About Body and Health Questionnaire (Rief et al., 2018), checklist of relationship to medicine and compliance.

**Results:**

Moderation analysis indicates that in people with higher hypochondriac beliefs negative news about alternative medicine lead to lower readiness to use these methods but also to comply with any medical recommendations (p<.01). In people with higher hypochondriac beliefs negative news about traditional medicine decrease readiness to use it but not alternative medicine (p<.01).

**Conclusions:**

Negative news about formal medicine situationally decrease readiness to use it while negative news about alternative medicine situationally decrease any readiness for treatment. Research is supported by the Russian Foundation for Basic Research, project No. 20-013-00799.

